# CSRQ: Communication-Efficient Secure Range Queries in Two-Tiered Sensor Networks

**DOI:** 10.3390/s16020259

**Published:** 2016-02-20

**Authors:** Hua Dai, Qingqun Ye, Geng Yang, Jia Xu, Ruiliang He

**Affiliations:** 1 School of Computer Science and Technology, Nanjing University of Posts and Telecommunications, No.66 Xinmofan Road, Nanjing 210013, China; 1214043108@njupt.edu.cn (Q.Y.); yangg@njupt.edu.cn (G.Y.); xujia@njupt.edu.cn (J.X.); 13041015@njupt.edu.cn (R.H.); 2 Key Laboratory of Broadband Wireless Communication & Sensor Network Technology, Nanjing University of Posts and Telecommunications, Ministry of Education, Nanjing 210013, China

**Keywords:** two-tiered sensor networks, range query, privacy and integrity preserving, encrypted constraint chain

## Abstract

In recent years, we have seen many applications of secure query in two-tiered wireless sensor networks. Storage nodes are responsible for storing data from nearby sensor nodes and answering queries from Sink. It is critical to protect data security from a compromised storage node. In this paper, the Communication-efficient Secure Range Query (CSRQ)—a privacy and integrity preserving range query protocol—is proposed to prevent attackers from gaining information of both data collected by sensor nodes and queries issued by Sink. To preserve privacy and integrity, in addition to employing the encoding mechanisms, a novel data structure called encrypted constraint chain is proposed, which embeds the information of integrity verification. Sink can use this encrypted constraint chain to verify the query result. The performance evaluation shows that CSRQ has lower communication cost than the current range query protocols.

## 1. Introduction

**Figure 1:**
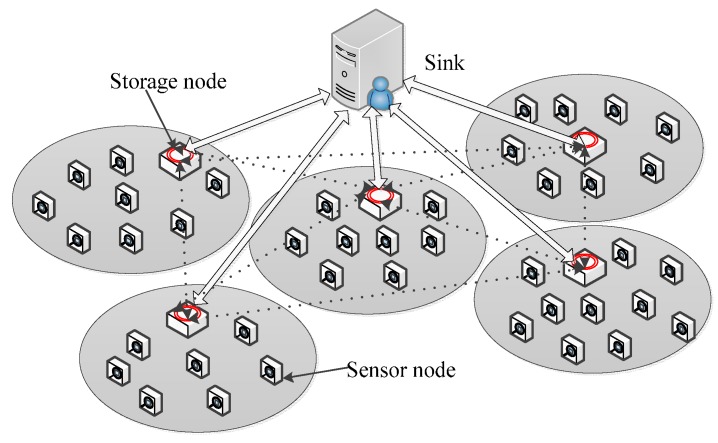
Architecture of two-tiered Wireless Sensor Networks.

Wireless sensor networks (WSNs) provide effective and convenient solutions for various applications, such as environment sensing, military target tracking, intelligent transportation system, *etc*. Two-tiered wireless sensor network is a kind of practical WSN, and its architecture is illustrated in Figure 1 [1,2]. The lower tier of two-tiered WSNs is composed of massively sensor nodes with limited storage and energy that are responsible for collecting data items, while the upper tier is composed of fewer resource-rich storage nodes, whose main tasks are storing data submitted by the nearby sensor nodes and answering queries issued by Sink. Compared to traditional wireless sensor networks, two-tiered WSNs have some significant advantages by interposing storage nodes as an intermediate tier. Firstly, the network topology of two-tiered WSNs is simpler. Secondly, two-tiered WSNs have a higher efficiency on query processing because Sink only communicates with storage nodes for queries.

However, it brings some security challenges to sensor networks where the storage nodes serve as an intermediate tier between the sensor nodes and Sink. Storage nodes not only receive information from the nearby sensor nodes, but also answer queries issued by Sink. Thus, the storage nodes are more attractive to attackers in two-tiered WSNs. Once it is compromised, the sensitive information stored in storage node will be obtained or guessed by the attackers, and the compromised storage node will make the query result incorrect or incomplete by maliciously inserting, deleting or tampering with the information. Therefore, it is important to study how to protect both the privacy of sensory data and integrity of the query result, even if the storage node is compromised.

Range query is an important type of query in sensor networks. In this paper, we focus on the secure range query processing. The security goals of secure range query include: (1) preserving the privacy of sensory data items and the interested range issued by Sink; and (2) preserving the integrity of the query result. There are two challenges that need to be addressed during the process to achieve these goals above: one is how to process queries without knowing the exact values of data items in sensor networks, and the other is how to verify whether or not the query result is correct and complete.

Therefore, we propose a communication-efficient secure range query processing method, denoted as CSRQ, which is a novel protocol for preserving the privacy and integrity of range query. When deployed in a hostile environment, we use a new data structure named Encrypted Constraint Chain to submit sensory data to the storage nodes. It ensures that the storage nodes cannot disclose the data stored on them. In addition, the message embedded in this chain makes the juggled and/or incomplete data items in queries detectable.

The main contributions in this paper are as follows: (1) A novel encrypted constraint chain model is proposed. Data items are submitted with a complete encrypted chain, which can preserve the privacy of sensitive data in sensor networks. Furthermore, adjacent relations of factors are embedded in chains, which can be used to verify the integrity of query result; (2) Based on the encrypted constraint chain model, a new scheme named CSRQ is proposed. CSRQ can protect sensitive data in two-tiered WSNs from the compromised storage nodes and allow Sink to verify whether the query result is complete and correct; (3) We evaluate our solutions by comprehensive simulation based on real datasets, and the results show that our scheme has a better performance on communication cost.

The rest of this paper is organized as follows. Section 2 gives a brief review of related works. Section 3 describes the system model and attack model. Section 4 proposes the scheme of encrypted constraint chain. Section 5 introduces the process of our protocol in detail. Section 6 analyzes the performance of our approach. We evaluate our approach using thorough experiments in Section 7 and conclude this paper in Section 8.

## 2. Related Works

Secure queries in two-tiered WSNs have drawn wide attention recently. Prior solutions for secure query in two-tiered WSNs include Top-*k* queries [3,4,5,6,7,8,9], MAX/MIN queries [10,11], range query processing, data aggregation in [12], k-NN processing in [13], *etc*.

The processes of security range queries have been investigated in [14,15,16,17,18,19,20,21,22,23,24]. In [14], Sheng and Li, which is described as S&L below, employed the bucket partition idea to preserve privacy and an authentication encoding mechanism to verify the integrity of query result in two-tiered WSNs. The basic idea is to divide the domain of data values into multiple buckets and distribute data items into these buckets. In each time slot, the sensor nodes encrypt data items together in each bucket and send them along with bucket ID to the nearby storage node. Then, Sink finds the minimal set of bucket IDs that contains the range in query, and sends the set as the query to storage. The storage nodes find encrypted data items in these buckets and send them to Sink. Finally, Sink decrypts the encrypted buckets. However, the communication cost and memory cost will increase exponentially with increase of the number of buckets in this scheme. In [15,16], Shi *et al.* proposed an optimized version of S&L’s scheme to reduce the communication cost between the sensor nodes and storage nodes. The main contribution of their optimization is that a new spatiotemporal crosscheck approach is proposed to verify the integrity of query result, which reduces the communication costs.

However, there are two main drawbacks existing in the schemes of both S&L and Shi *et al*, which are inherited from the bucket partitioning technique. (1) The compromised storage nodes could obtain reasonable estimation of actual values of both data items and queries [17]; (2) The communication cost increases exponentially with the number of dimensions of collected data. For the sake of these problems, Chen and Liu proposed SafeQ in [17,18], which has a better safety performance in preserving the privacy and integrity of range queries. The basic idea of SafeQ is that a prefix-encoding scheme is proposed to encode both data items and queries such that it could not be estimated by compromised storage nodes, and a new data structure called neighborhood chain is proposed to generate integrity verification information, and Sink can verify the integrity of query result using this information.

Although the prefix-encoding scheme and neighborhood chains structure proposed in SafeQ can solve the problems in S&L’s scheme, they will increase the memory and communication costs for both sensor nodes and storage nodes. The main reason is that each data item needs to be stored twice in the structure of neighborhood chains. Therefore, Yi *et al.* proposed a new link watermarking scheme named QuerySec in [19], a protocol based on two new techniques: (1) a scheme based on order preserving function for preservation of data privacy; and (2) a new link watermarking scheme for verification of query results. In [21], Nguyen *et al.* proposed a novel model based on a *d-*disjunct matrix, an order-preserving function and a permutation function to preserve the privacy of sensitive information for the range queries, while it fails to consider the verification of query result. 

In [22], an efficient secure range query protocol named ESRQ is proposed to realize more efficient and correct process of range query. In [23], Dong and Zhang provided an extend version of [22], and they were the first ones to focus on collusion attacks for range queries in two-tiered WSNs. The basic idea is that different sensor nodes have different hash functions to encode data items for the protection of data privacy and the correlation among data is used for verification of result. In [24], Dong and Chen *et al.* proposed SecRQ, which not only protects the privacy of data, but also consider the collusion attacks and probability attacks in two-tiered WSNs. It adopts generalized inverse matrices and distance-based range query mechanism for the security of data. Besides, a mutual verification scheme is proposed to verify the integrity of query results in this paper, and it verifies the integrity of query result with lower false positive rate and lower communication cost than the schemes mentioned above.

## 3. Models and Problems Statement

### 3.1. Network Model

The adopted architecture of two-tiered WSNs is shown in Figure 1, which is similar to [17]. Two-tiered WSN is a special kind of wireless sensor network, in which the storage node *M* is the intermediate tier of networks, and the lower tier is composed of sensor nodes. The whole network is divided into a number of *cells*. Each *cell* consists of *M* and a number of sensor nodes *S* = {*s*_1_, *s*_2_, …, *s_n_*}, which can be denoted as *cell =* {*M*,{*s*_1_, *s*_2_, …, *s_n_*}}. Sensor nodes are inexpensive sensing devices with limited storage and energy resource and are in charge of collecting data items from a *cell* and submitting information of data items to nearby *M*. *M* is resourceful device with relatively high storage and energy resources and is responsible for receiving and storing information submitted by nearby sensor nodes and answering queries issued by Sink. Sink gathers query results from multiple *M* and computes the final query result. 

### 3.2. Query Model

The range query refers to accessing all the sensory data items included in a specified range, which can be denoted as a three-tuple:

*Q_t_* = (ψ, *t*, [*low*, *high*])

where ψ denotes the set ID of queried sensor nodes, *t* is the queried time slot, and *low* and *high* refer to the lower and upper bounds of query range, respectively. For example, if *Q_t_* = ({*s*_1_, *s*_2_, …, *s*_8_}, *t*, [10,20]), it means that Sink finds all data items in [10,20] collected by sensor nodes *s*_1_–*s*_8_ during a time slot *t*.

For the sake of brevity, we only discuss the range query that Sink queries in a *cell* *=* {*M*, {*s*_1_, *s*_2_, …, *s_n_*}} during time slot *t* in this paper, which can be denoted as *Q_t_* = (ψ, *t*, [*low*, *high*]), where ψ = {*s*_1_, *s*_2_, …, *s_n_*}. To get the results of queries in multiple time slots and/or multiple cells, we can simply get the final result by resolving and merging the single results.

### 3.3. Threat Model

By using the same threat model in [17,18,19,20,21,22], we assume that Sink and the sensor nodes are trusted in two-tiered WSNs, but *M* is not. In fact, the sensor nodes can also be compromised in a hostile environment. Attackers may obtain sensitive data items from compromised sensor nodes. A sensor node only contains a small fraction of data items collected by all sensor nodes, while a great deal of sensitive data items are stored in *M*. Therefore, attackers can steal less information from a compromised sensor node than from *M*. Therefore, we are mainly concerned with the scenario of the compromised *M* in this paper.

If *M* is compromised in two-tiered WSNs, we consider that attackers can attack networks in the following two ways:

(1) The attackers obtain sensitive information stored in *M* directly or indirectly, which violates the privacy of data.
(2) The attackers forge or exclude the legitimate data items stored in compromised *M*, which makes the query result incorrect or incomplete.

### 3.4. Problems Statement

The goal of secure range query is not only to preserve the privacy of data items collected by sensor nodes and queries issued by Sink, but also to ensure that the integrity of query result can be verified by Sink. The details are as follows:

(1) The privacy issues: *M* could not obtain the actual values of any data items collected by sensor node and the values of lower and higher bounds of query range in *Q_t_*.
(2) The integrity issues: If *Q_t_* = (ψ, *t*, [*low*, *high*]), the query result, which can be denoted as *QR*, should contain all data items satisfying [*low*, *high*] in ψ. All data items in *QR* are collected by the sensor nodes in ψ.

The keys to achieve the security goals above are as follows. First, *M* could decide whether a data item collected by sensory node should be included in query result by comparing it with *low* and *high* without knowing the actual values of them. Second, Sink could detect whether all data items satisfying [*low*, *high*] are included in *QR* and whether all data items included in *QR* satisfy [*low*, *high*], and that all of them are collected by the sensor nodes in ψ. Thus, we propose CSRQ, a query protocol with better performance in terms of both security and communication cost in preserving the privacy of data items and the integrity of query results.

Moreover, as an index used to evaluate the performance of security range query protocol, the communication costs include two aspects: one is the communication cost for sensor node, which plays a decisive role in the lifecycles of sensor networks; the other is the communication cost between *M* and Sink, which directly affects the operating costs of networks. We conduct a detailed analysis for the index of performance in Section 6 and Section 7.

## 4. Encrypted Constraint Chain Model

In this section, we will introduce the encrypted constraint chain in detail, which is proposed to protect the privacy and integrity of two-tiered WSNs.

**Definition 1.**  Encrypted constraint chain: Given n numbers stored in the ascending order D = {d_1_, d_2_, …, d_n_}, where d_1_ < … < d_n_, we partition these numbers into several parts with parameter τ, and encrypt every part. These encrypted parts can easily be brought together to form encrypted constraint chain C_τ_.

*C_τ_* = *F*_1_⋈*F*_2_⋈...⋈*F_δ_* (1)

Here “⋈” ⋈denotes concatenation, and *δ* is the number of items in *C_τ_*. We call *F_i_* the constraint factor of *C_τ_*, and *F_i_.ds* represents the dataset in *F_i_*. *C_τ_* satisfies following conditions:

(1) *F_i_* has *τ* sensory data items, where 1 ≤ *i* ≤ *δ* − 1, while *F_δ_* has no more than *τ* sensory data items.
(2) The upper and lower bounds of *F_i_* are denoted as *UB*(*F_i_*) and *LB*(*F_i_*), respectively. Hence, for any two adjacent constraint factors *F_i_* and *F_i_*_+1_, we can determine *UB*(*F_i_*) = *LB*(*F_i_*_+1_).
(3) The computation formula of *δ* is
  (2) $$\delta =\left\{ \begin{array}{c} 1\;\tau \geq n\\ 1+\left\lceil \frac{n-\tau }{\tau -1}\right\rceil \;\tau <n \end{array}\right.$$

The form of *F_i_* in *C_τ_* is as shown in Equation (3), in which *k* represents an encryption key, and “||” denotes the concatenation of data items.

(3) $$F_{i}=\left\{ \begin{array}{l} {\left(d_{1+(i-1)\cdot (\tau -1)}||...||d_{1+i\cdot (\tau -1)}\right)}_{k}\;1\leq i<\delta \\ {\left(d_{1+(\delta -1)\cdot (\tau -1)}||...||d_{n}\right)}_{k}\;i=\delta \end{array}\right.$$

**Definition 2.**  *Let C_τ_ = F_1_*⋈*F_2_*⋈*...*⋈*F_δ_ be an encrypted constraint chain, where δ is called the length of C_τ_ and it is denoted as |C_τ_| = δ. F_i_ satisfies that F_i_* ∈ *C_τ_, and F_i-1_ and F_i+1_ are called the left and right neighbor constraint factors of F_i_, respectively. The head factor of C_τ_ is denoted as head(C_τ_) = F_1_ and the tail factor is denoted as tail(C_τ_) = F_δ_*.

**Definition 3.**  *Given two encrypted chains C_τ_ and C_τ_’. If each factor of C_τ_’ is included in C_τ_, then C_τ_’ is called a sub-chain of C_τ_. It can be denoted as C_τ_’* ⊆ *C_τ_. Thus, we have*

(4) $$\forall F_{i}\in {C_{\tau }}^{\prime }\left(F_{i}\in C_{\tau }\right)\Rightarrow {C_{\tau }}^{\prime }\subseteq C_{\tau }$$

According to Definition 3, we can easily deduce the following property.

**Property 1.**  *The sub-chain relation* ⊆ *is transitive, which means*

(5) $${C_{\tau }}^{\prime \prime }\subseteq {C_{\tau }}^{\prime }\wedge {C_{\tau }}^{\prime }\subseteq C_{\tau }\Rightarrow {C_{\tau }}^{\prime \prime }\subseteq C_{\tau }$$

**Definition 4.**  *Given an encrypted constraint chain C_τ_, let C_τ_’ be a sub-chain of C_τ_, which means C_τ_’* ⊆ *C_τ_. For a query range* [*low, high*]*, if C_τ_’ simultaneously satisfies the following conditions, then C_τ_’ is called a Maximum Encrypted Constraint Sub-chain (MECS) of C_τ_*.

(1) $LB(head({C_{\tau }}^{\prime }))<low\leq UB(head({C_{\tau }}^{\prime }))\wedge LB(tail({C_{\tau }}^{\prime }))\leq high<UB(tail({C_{\tau }}^{\prime }))$
(2) $\forall F_{i}\in ({C_{\tau }}^{\prime }-head({C_{\tau }}^{\prime })-tail({C_{\tau }}^{\prime }))\left(\forall d_{j}\in F_{i}.ds(d_{j}\in [low,high])\right)$
(3) $\forall F_{i}\in C_{\tau }\wedge F_{i}\notin {C_{\tau }}^{\prime }\left(\forall d_{j}\in F_{i}.ds\left(d_{j}\notin [low,high]\right)\right)$

Given a *MECS* of *C_τ_* that satisfies [*low*, *high*], according to *Definition* 4, we can know that *low* is between the lower and upper bounds of head constraint factor in *MECS*, and *high* is between the lower and upper bounds of tail constraint factor. Except the head and tail, the data items in each factor of *MECS* are between *low* and *high*, and the data items included in *C_τ_* but outside of *MECS* are not included in [*low*, *high*].

Now we give a further instruction of the above definitions and properties with some examples. For example, *D* = {3, 6, 11, 23, 38, 42}. Given a parameter *τ* = 3, the encrypted constraint chain is *C_τ_* = (3||6||11)*_k_*⋈(11||23||38)*_k_*⋈(38||42)*_k_*, where |*C_τ_*| = 3, *UB*((3||6||11)*_k_*) = *LB*((11||23||38)*_k_*) and *UB*((11||23||38)*_k_*) = *LB*((38||42)*_k_*). Given a range [7,23], the *MECS* of *C_τ_* which satisfies [7,23] is *C_τ_’ =* (3||6||11)*_k_*⋈(11||23||38)*_k_*.

As demonstrated by the previous definitions and properties, if *F_i_* and *F_i+_*_1_ are two adjacent factors in the encrypted constraint chain, the upper bound of *F_i_* equals to the lower bound of *F_i+_*_1_. Thus, it provides a theoretical basis for the integrity verification of query results. 

## 5. Secure Range Query Protocols

In this paper, we employ the 0-1 encoding mechanism [25] to preserve privacy. The basic idea of 0-1 encoding mechanism is to convert the verification of whether a data item is within a range to the verification of whether there are intersections between two sets. Given a number *x* whose binary format is *b*_1_*b*_2_*…b_n_*_−1_, *b_n_* ∈ {0,1}*^n^*, where *n* is the bit length of number *x*. The 0-coding of *x* is defined as *E*^0^(*x*) = {*b*_1_*b*_2_…*b_i_*_−1_|*b_i_* = 0 ∧ 1 ≤ *i* ≤ *n*} and the 1-coding of it is defined as *E*^1^(*x*) = {*b*_1_*b*_2_…*b_i_*|*b_i_* = 1 ∧ 1 ≤ *i* ≤ *n*}. If and only if *E*^1^(*x*) ∩ *E*^0^(*y*) ≠ Ø, *x* > *y*, else, *x* ≤ *y*. According to the above definitions, given two numbers *x* and *y*, they can be compared with each other using their 0-1 codings. What is noteworthy is that *x* and *y* can be compared only if they are of different encoding types, which means that they have the encoding types of 0-coding and 1-coding, respectively.

In this paper, we convert each 0-1 coding to a corresponding unique number using the numerical function 𝒩(*) similarly to that used in [18] and we encode the 0-1 coding data items using the keyed-Hash Message Authentication Code (HMAC) to ensure that it is infeasible for *M* to steal sensitive data items. We denote HMAC function as *HMAC_g_*(*), where *g* is a key for HMAC which is only known to sensor node and Sink. A data processed by 0-1 encoding and HMAC is denoted as a comparator. *HNE*^0^(*x*) = *HMAC_g_*(𝒩(*E*^0^(*x*))) is a comparator of 0-coding and *HNE*^1^(*x*) = *HMAC_g_*(𝒩(*E*^1^(*x*))) is a comparator of 1-coding. Thus, we can easily know *x* > *y* if and only if *HNE*^1^(*x*) ∩ *HNE*^0^(*y*) ≠ Ø.

### 5.1. Submission Protocol

The submission protocol concerns how a sensor node submits its data to the nearby *M*. First, the sensor node encrypts all the data items collected during a time slot *t*, then builds the corresponding encrypted constraint chain and computes the comparators of encrypted data items. After that, the sensor node sends the encrypted constraint chain to the nearby storage node.

We denote *d_max_* and *d_min_* as the lower and upper bounds of a sensory data item, respectively. Let *τ* be the partition parameter of encrypted constraint chain. Each sensor node *s_i_* in a network shares a secret key *k_i,t_* with Sink. The submission protocol is illustrated as the following Protocol 1.

**Protocol 1: Submission Protocol** Let *d_i_*_,1_, *d_i_*_,2_, …, *d_i,N_* be *N* data items collected by *s_i_* during time slot *t*. For simplicity, we assume *d_min_* ≤ *d_i_*_,1_ ≤ … ≤ *d_i_*_,*N*_ ≤ *d_max_*. Let *D_i_* = {*d_min_*, *d_i_*_,1_, …, *d_i_*_,*N*_, *d_max_*}. Then *s_i_* performs the following steps. (1) Compute the 0-1 code of each data item. (2) Build the encrypted constraint chain *C_τ_*_,*i*_ of *D_i_* with *τ* and *k_i_*_,*t*_. Assuming *C_τ_*_,*i*_ = *F_i_*_,1_⋈*F_i_*_,2_⋈...⋈*F_i_*_,*δ*_, we can compute *δ* and *F_i_*_,*j*_ as follows.(6)$$\delta =\left\{ \begin{array}{c} 1\;\tau \geq N+2\\ 1+\left\lceil \frac{N+2-\tau }{\tau -1}\right\rceil \;\tau <N+2 \end{array}\right.$$
(7)$$F_{i,j}=\left\{ \begin{array}{ll} {\left(d_{\min}||d_{i,1}||...||d_{i,\tau -1}\right)}_{k_{i,t}} & j=1\\ {\left(d_{i,(j-1)\cdot (\tau -1)}||...||d_{i,j\cdot (\tau -1)}\right)}_{k_{i,t}} & 1<j<\delta \\ {\left(d_{i,(\delta -1)\cdot (\tau -1)}||...||d_{n}||d_{\max}\right)}_{k_{i,t}} & j=\delta \end{array}\right.$$ (3) Compute the comparator of *UB*(*F_i_*_,*j*_), where 1 ≤ *j* ≤ *δ* − 1. It means computing *HNE*^0^(*UB*(*F_i_*_,*j*_)) and *HNE*^1^(*UB*(*F_i_*_,*j*_)). Then, add the comparator set which contains the fewest elements into Ω*_i_*. Thus we have,

Ω*_i_* = {*min*{*HNE*^0^(*UB*(*F_i_*_,*j*_)), *HNE*^1^(*UB*(*F_i_*_,*j*_)) | *F_i_*_,*j*_ ∈ *C_τ_*_,*i*_ ∧ 1 ≤ *j* ≤ *δ* − 1}}
(8)
where *min*(*X*, *Y*) denotes the set containing the fewest elements. (4) Send the following message to *M*, where *id*(*s_i_*) denotes the ID of *s_i_* in networks.
*s_i_* → *M*: <*id*(*s_i_*), *t*, *C_τ_*_,*i*_, Ω*_i_*>


In order to build the encrypted constraint chain easily, we denote an encrypted group as a constraint factor in this paper. Thus, the more sensory data are contained in an encrypted group, the less encrypted data and HMAC data are contributed by a sensor node. It contains at most ⌊lew⌋ sensory data items in an encrypted group, where the length of sensory data items is *w* and the length of an encrypted group is *l_e_*. Thus, we can set *τ =* ⌊lew⌋ to reduce the communication cost consumed by the data submitting of sensor nodes.

### 5.2. Query Protocol

The query protocol concerns how Sink and *M* process the queries correctly. To ensure that both the privacy of data and the integrity of query result can be preserved, we will give the basic idea of our query protocol. First, Sink computes the comparators of *low* and *high* in *Q_t_* = (ψ, *t*, [*low*, *high*]), and then replaces the *low* and *high* in *Q_t_* with their comparators respectively. After that, Sink sends the modified *Q_t_* to *M*. Second, after receiving a query, *M* decides whether the value of comparator in encrypted constraint chain contributed by sensor node is included in [*low*, *high*] using the 0-1 coding mechanism, and computes the minimal set which contains all data items satisfying the queries. Then, *M* sends this set to Sink. Third, after receiving this minimal set, Sink decrypts each encrypted data in dataset, and computes the *QR*. Finally, Sink verifies the authenticity and completeness of *QR*. The above steps show the basic idea of query protocol, and the detailed performance of query is as follows:
**Protocol 2: Query Protocol****Phase 1: Sink sends query to *M*** Sink firstly computes {*HNE*^0^(*low*), *HNE*^1^(*low*), *HNE*^0^(*high*), *HNE*^1^(*high*)}, the comparator of *low* and *high* in *Q_t_* = (ψ, *t*, [*low*, *high*]), and replaces the *low* and *high* in *Q_t_* with their corresponding comparators, respectively. Then, Sink sends *Q_t_* = (ψ, *t*, {*HNE*^0^(*low*), *HNE*^1^(*low*), *HNE*^0^(*high*), *HNE*^1^(*high*)}) to *M*. **Phase 2: *M* processes the query** Upon receiving *Q_t_* from Sink, *M* performs the following two steps. We denote *CS* as the minimal encrypted dataset received by Sink, which contains the query results. The initial *CS* is empty, which is denoted as *CS* = Ø. (1) Let *C_τ_*_,*i*_ = *F_i_*_,1_⋈*F_i_*_,2_⋈...⋈*F_i_*_,δ_ be an encrypted constraint chain contributed by *s_i_* during a time slot, and *min*{*HNE*^0^(*UB*(*F_i_*_,*j*_)), *HNE*^1^(*UB*(*F_i_*_,*j*_))} is the comparator of *UB*(*F_i_*_,*j*_), which is a factor in Ω*_i_*. According to *Definition* 1, *LB*(*F_i_*_,*j*_) = *UB*(*F_i_*_,*j*−1_) (1 < *j* ≤ *δ*), it is clear that the comparator of *LB*(*F_i_*_,*j*_) is *min*{*HNE*^0^(*UB*(*F_i_*_,*j*−1_)), *HNE*^1^(*UB*(*F_i_*_,*j*−1_))} in Ω*_i_*. Here, the 0-1 encoding technology is employed to compare *UB*(*F_i_*_,*j*_) and *LB*(*F_i_*_,*j*_) with *low* and *high*, where *F_i_*_,*j*_ is a constraint factor in *C_τ_*_,*i*_. If one of the following three conditions can be satisfied, add *F_i_*_,*j*_ into ${ℑ}_{i}$, where ${ℑ}_{i}$ is a set of constraint factor. We set ${ℑ}_{i}$ = Ø initially. **Condition 1:**
*low* ≤ *LB*(*F_i_*_,*j*_) ≤ *high* **Condition 2:**
*low* ≤ *UB*(*F_i_*_,*j*_) ≤ *high* **Condition 3:**
*LB*(*F_i_*_,*j*_) ≤ *low* ∧ *high* ≤ *UB*(*F_i_*_,*j*_) After all constraint factors in *C_τ_*_,*i*_ are processed through the above steps, then all the constraint factor set ${ℑ}_{i}$ will be added into another set *CS*. (2) After all sensor nodes are processed through step (1), *M* will send the following message to Sink.
*M→* Sink: <*t*, {*id*(*s_i_*), *ℑ_i_* | *s_i_* ∈ ψ ∧ *ℑ_i_* ⊆ *CS* }>

**Phase 3: Sink receives the message** After receiving the message from *M*, Sink decrypts the encrypted message in *CS* and computes the query result *QR*, and then verifies the integrity of *QR.* The process of verification is detailed in Algorithm 1 of Section 5.3.

The Protocol 2 shows that the *CS* received by Sink is as follows:

(9) $$CS=\cup_{s_{i}\in \Psi }\{{ℑ}_{i}\}$$

**Property 2.**  *In CS, it includes at least one item in* ${ℑ}_{i}$ *contributed by s_i_* ∈ ψ, *so we have*

(10) $$\forall s_{i}\in \Psi \to |{ℑ}_{i}|\geq 1$$

**Proof:**  Let *C_τ_*_,*i*_ = *F_i_*_,1_⋈*F_i_*_,2_⋈...⋈*F_i_*_,*δ*_ be a *MECS* received by *M* from *s_i_*. According to *Definition* 1, we can easily know that any constraint factor’s upper bound equals to the next factor’s lower bound, which means *UB*(*F_i_*_,*j*_) = *LB*(*F_i_*_,*j*+1_). [*LB*(*F_i_*_,*j*_), *UB*(*F_i_*_,*j*_)] is a range interval composed of the lower and upper bounds of *F_i_*_,*j*_. Thus, the composition process of *C_τ_*_,*i*_ in Protocol 1 shows that the following Equation (11) is true.

(11) $$\left[d_{\min},d_{\max}\right]=\cup_{1\leq j\leq \delta }\left[LB(F_{i,j}),UB(F_{i,j})\right]$$

Because *d_min_* and *d_max_* are the lower and upper bounds of sensory data, the query range [*low*, *high*] must be included in [*d_min_*, *d_max_*]. According to this, we know that there is at least one constraint factor *F_i_*_,*j*_ that satisfies [*LB*(*F_i_*_,*j*_),*UB*(*F_i_*_,*j*_)] ∩ [*low*, *high*] ≠ Ø. There exist the following four possible cases.

**Case 1.** *LB*(*F_i_*_,*j*_) ≤ *low* ≤ *UB*(*F_i_*_,*j*_) ≤ *high*
**Case 2.** *LB*(*F_i_*_,*j*_) ≤ *low* ≤ *high* ≤ *UB*(*F_i_*_,*j*_)
**Case 3**. *low* ≤ *LB*(*F_i,j_*) ≤ *high* ≤ *UB(F_i,j_)*
**Case 4**. *low* ≤ *LB*(*F_i,j_*) ≤ *UB*(*F_i,j_*) ≤ *high*

According to Protocol 2, it is easy to know that *F_i_*_,*j*_ should be added into ${ℑ}_{i}$ if any one of the above four cases is satisfied. Thus, there is at least one item included in ${ℑ}_{i}$, which means Property 2 is true.

**Property 3.**  *We assume C_τ,i_ is an encrypted constraint chain that M receives from s_i_* ∈ ψ*. Thus* ${ℑ}_{i}$ *in Protocol 2 is a MECS of* *C_τ,i_ which satisfies* [*low, high*].

**Proof:**  Assume that ${ℑ}_{i}$ = {*F_i_*_,*j*_, *F_i_*_,*j*+1_, …, *F_i_*_,*j*+*p*−1_}, where *p* ≥ 1. Based on the three conditions that must be satisfied in *MECS* of Definition 4, we give a proof of Property 3. (1) According to the process of forming ${ℑ}_{i}$ in Protocol 2, if *F_i_*_,*j*−1_ and *F_i_*_,*j*_ are included in *C_τ_*_,*i*_, *UB*(*F_i_*_,*j*−1_) < *low*. If *UB*(*F_i_*_,*j*−1_) ≥ *low*, *F_i_*_,*j−*1_ should also be added into ${ℑ}_{i}$. Thus, it would be contradictory to the assumption that ${ℑ}_{i}$ = {*F_i_*_,*j*_, *F_i_*_,*j*+1_, …, *F_i_*_,*j*+*p*−1_}. What is more, Definition 1 shows that *UB*(*F_i,j_*_−1_) = *LB*(*F_i,j_*). If *F_i,j_* ∈ ${ℑ}_{i}$, then *LB*(*F_i,j_*) < *low ≤ UB*(*F_i,j_*). Similarly, if *F_i_*_,*j*+*p*_ and *F_i_*_,*j*+*p*−1_ are included in *C_τ,i_*, *LB*(*F_i_*_,*j*+*p*_) ≤ *high < UB*(*F_i_*_,*j*+*p*_). Furthermore, *head*(${ℑ}_{i}$) = *F_i,j_* and *tail*(${ℑ}_{i}$) = *F_i,j_*_+*p*_ show that ${ℑ}_{i}$ satisfies the first condition of Definition 4. (2) The conclusion that *LB*(*F_i,j_*) < *low ≤ UB*(*F_i,j_*) and *LB*(*F_i_*_,*j*+*p*_) ≤ *high < UB*(*F_i_*_,*j*+*p*_) in (1) indicate that any factors between *F_i_*_,*j*_ and *F_i_*_,*j*+*p*_ are included in [*low*, *high*], and the values of data in factors before *F_i_*_,*j*_ are smaller than *low*, and those in factors after *F_i_*_,*j+p*_ are bigger than *high*. It means that factors out of ${ℑ}_{i}$ are not included in [*low*, *high*]. Thus, it illustrates that ${ℑ}_{i}$ can also satisfy Conditions 2 and 3 in Definition 4.

Based on (1) and (2), it can be proven that ${ℑ}_{i}$ is the *MECS* of *C_τ_*_,*i*_ which satisfies [*low*, *high*].

**Theorem 1.**  *CS is the minimal encrypted dataset, which includes all data items satisfying* [*low, high*]*, where CS is contributed by sensor nodes in* ψ.

**Proof:**  Because all of ${ℑ}_{i}$ contributed by *s_i_* ∈ ψ are *MECS* of *C_τ,i_* that satisfy [*low*, *high*], according to the definition of *MECS*, we can know that any data items in constraint factors of ${ℑ}_{i}$ are included in [*low*, *high*], and any data items out of ${ℑ}_{i}$ are not included in [*low*, *high*]. Thus, ${ℑ}_{i}$ is the minimal encrypted dataset which includes all data items satisfying [*low*, *high*] in *C_τ_*_,*i*_. As shown in Equation (9), $CS=\cup_{s_{i}\in \Psi }\{{ℑ}_{i}\}$, therefore, *CS* is the minimal encrypted dataset which satisfies [*low*, *high*] in ψ.

### 5.3. The Computation of Query Result and the Algorithm of Integrity Verification

After receiving *CS* from *M*, Sink will decrypt the encrypted data in *CS* using the keys shared with sensor nodes and compute the query result *QR*, and then, it will verify the integrity of *QR*. Algorithm 1 shows the details of integrity verification.

**Algorithm 1:** The algorithm of integrity verificationLet $CS=\cup_{s_{i}\in \Psi }\{{ℑ}_{i}\}$ be an encrypted dataset that Sink receives from *M*. Sink verifies the integrity of *QR* as follows. Sink performs the following three steps to verify each ${ℑ}_{i}$ contributed by *s_i_*
∈ ψ. (1) If ${ℑ}_{i}$ = Ø, it could not satisfy Definition 2. Thus the integrity of *QR* is violated. Quit the algorithm. (2) If ${ℑ}_{i}$ ≠ Ø, Sink decrypts all factors in ${ℑ}_{i}$ using *k_i_*_,*t*_ only shared with *s_i_*, and checks whether both of following two conditions are satisfied. If so, add all the data within [*low*, *high*] into *QR*, and then turn to Step (3). Otherwise, the integrity of *QR* is violated so quit the algorithm.  1) Each factor *F_i_*_,*v*_ in ${ℑ}_{i}$ satisfies following condition:
*low* ≤ *LB*(*F_i_*_,*v*_) ≤ *high* ∨ *low* ≤ *UB*(*F_i_*_,*v*_) ≤ *high* ∨ *LB*(*F_i_*_,*v*_) ≤ *low* ∧ *high* ≤ *UB*(*F_i_*_,*v*_)
  2) *F_i_*_,*k*_ and *F_i_*_,*v+*1_ satisfy the following formula, where *F_i_*_,*v*_ and *F_i_*_,*v+*1_ are two adjacent factors in ${ℑ}_{i}$.
*UB*(*F_i_*_,*v*_) = *LB*(*F_i_*_,*v*+1_)

(3) If all factors contributed by *s_i_*
∈ ψ are processed through Steps (1) and (2), and all of them satisfy the query, then the *QR* satisfies the query, thus return the *QR*. Otherwise, continue to process the next ${ℑ}_{i}$ contributed by unprocessed sensor node, and turn to Step (1).

Algorithm 1 shows that the key to verify the integrity of the *QR* is to check whether all of following three conditions can be satisfied. First, each sensor node *s_i_* to be queried contributes a non-empty set ${ℑ}_{i}$. Second, all factors in *QR* satisfy the query range [*low*, *high*]. Third, the upper bound of each factor equals to the lower bound of next one. If and only if all of the above three conditions are satisfied will *QR* satisfy the integrity of query results.

## 6. Protocol Analyses

In two-tiered WSNs, we mainly evaluate the performance of a security query protocol from following two aspects: one is security, and the other is the communication cost. In this section, we will analyze the performance of CSRQ from these two aspects.

### 6.1. Security Analysis

#### 6.1.1. Privacy Analysis

(1) The privacy of sensory data. The key to preserve the privacy of data items in two-tiered WSNs is to ensure that *M* cannot steal the actual values of encrypted data items without knowing the secret keys. If a storage node is compromised, CSRQ can effectively preserve the privacy of sensitive data. Because *s_i_* encrypts the collected data items using its private keys only shared with Sink before sending data items to *M* in CSRQ, it is very difficult for attackers to obtain the actual values of sensory data. Furthermore, the HMAC mechanism is employed in CSRQ to ensure that it is computationally infeasible to compute the actual values of sensory data without knowing both the Hash key and its secret key. Therefore, CSRQ has a better performance in protecting the privacy of sensory data.

(2) The privacy of query result. Similar to the privacy protection of sensory data, the key to protect privacy of query result is to ensure that *M* cannot get the actual values of results. In CSRQ, the sensor nodes send data items to *M* with the form of encrypted constraint chain and their corresponding comparators, and *M* compares the query range with data items without knowing the actual value of them, all data items included in *CS* are encrypted. Upon receiving *CS*, only Sink can decrypt the data items in *CS* and compute the query result. Therefore, without knowing the key used in the encryption, it is very difficult to steal the value of query result.

(3) The privacy of query range. In CSRQ, it does not allow attackers to obtain the actual values of query range either. Sink sends the query to *M* after replacing the query range with their corresponding comparators, which ensures that it is very difficult for *M* to leak the information of query range.

Thus, the CSRQ proposed in this paper can ensure that the privacy of sensory data, query result and query range can be protected.

#### 6.1.2. Integrity Analysis

In CSRQ, we propose a novel encrypted constraint chain to ensure that the integrity of query result can be verified by Sink. The main idea is that the data items collected by all sensor nodes during a time slot *t* will be sent to the nearby *M* with the form of a complete encrypted constraint chain, which allows Sink to verify the integrity of query result by checking the relationship of adjacent factors in the chain. The integrity verification includes the following two-fold: one is verifying whether the data item satisfying the query is forged, and the other is verifying whether the data items satisfying query are deleted by attackers.

Let ${ℑ}_{i}$ = *F_i,j_*⋈*F_i,j_*_+1_⋈…⋈*F_i,v_* be the *MECS* which satisfies *Qt* = (ψ, *t*, [*low*, *high*]) received by Sink from *s_i_* ∈ Ψ during time slot *t*. We assume that *M* is compromised and it attempts to attack ${ℑ}_{i}$. Next, we will analyze the integrity of CSRQ from the following cases. 

(1) If data item in *QR* satisfying the query is forged by the compromised *M*:

① If *F_i_*_,*u*_*’* is a tampered data item which replaces the original *F_i_*_,*u*_ in ${ℑ}_{i}$, Sink will find that *UB*(*F_i_*_,*u-*1_) ≠ *LB*(*F_i_*_,*u*_*’*) or *UB*(*F_i_*_,*u*_*’*) ≠ *LB*(*F_i_*_,*u*+1_) after decrypting ${ℑ}_{i}$. It could not satisfy the definition of encrypted constraint chain (*Definition* 1). Therefore, ${ℑ}_{i}$ can be determined as incomplete. Or Sink will detect that *d*_α_ ∈ *F_i_*_,*u*_*’*·*ds*, where *d*_α_ ∉ [*low*, *high*], which contraries to the definition of *MECS* (Definition 4). Thus *CR* can also be determined as incomplete.

② Similar to ① above, if *F_i_*_,*u*_*’* is inserted as a tampered data item between *F_i_*_,*u*_ and *F_i_*_,*u+*1_, Sink will detect that *UB*(*F_i_*_,*u*_) ≠ *LB*(*F_i_*_,*u*_*’*) or *UB*(*F_i_*_,*u*_*’*) ≠ *LB*(*F_i_*_,*u*+1_) after decrypting ${ℑ}_{i}$. It could not satisfy *Definition* 1 either, which means that *CR* is incomplete.

(2) If data item that satisfies the query range is deleted by *M*:

① If all data items in ${ℑ}_{i}$ are deleted by attackers, Sink will detect that ${ℑ}_{i}$ = Ø, which is contrary to Property 2. Thus, Sink can judge that ${ℑ}_{i}$ has been attacked.

② If the data items between *F_i_*_,*a*_ and *F_i_*_,*b*_ deleted by *M*, where *j ≤ a < b ≤ v*, Sink will detect that *UB*(*F_i_*_,*a*_) ≠ *LB*(*F_i_*_,*b*_), which dissatisfies Definition 1. Thus, ${ℑ}_{i}$ will be determined as incomplete.

③ If the head constraint factor of ${ℑ}_{i}$ *F_i_*_,*j*_ is deleted by attackers, *F_i_*_,*j+*1_ will be the new head(${ℑ}_{i}$) of ${ℑ}_{i}$. Then, Sink will detect that all data items in *F_i_*_,*j*+1_ are included in [*low*, *high*] after decrypting ${ℑ}_{i}$, which could not satisfy the Condition (1) of Definition 4. Therefore, the incomplete ${ℑ}_{i}$ can be detected by Sink. Similarly, if the deleted constraint factor *F_i_*_,*j*_ is tail (${ℑ}_{i}$), it can also be detected by Sink.

In conclusion, Sink can verify the correctness and completeness of query result effectively in CSRQ.

### 6.2. Communication Cost

#### 6.2.1. Communication Cost of Sensor Node

In two-tiered WSNs, the communication cost of sensor node is mainly incurred by transferring data items from the sensor nodes to *M*. Here, let *E_c_* be the communication cost during a data submission for each sensor node.

Let *n* be the size of two-tiered WSNs inquired about, and *l_t_* be the bit length of a time slot. We assume that each sensor node collects *N* data items during a time slot. According to the definition of encrypted constraint chain in our scheme, *N* data items will be divided into *δ* constraint factors by parameter *τ*, where *δ* can be calculated from Equation (2). Let *l_e_*, *l_h_* and *l_id_* be the average length of an encrypted constraint factor, a HMAC encoding and an ID of encrypted node, respectively, and *L* be the average hops from each sensor node to *M*. By analyzing Protocol 1, the formula to calculate the communication cost is gained as follows.

(12) $$E_{C}=\sum_{i=1}^{n}\left(l_{id}+l_{t}+\delta \cdot l_{e}+(\delta -1)\cdot l_{h})\right)\cdot L$$

#### 6.2.2. Communication Cost of Query

The query Protocol 2 shows that the query is a collaborative process between *M* and Sink, thus the communication costs of query should contain two aspects: one is the cost for sending the query from Sink to *M*, and the other is the cost for sending the message from *M* to Sink. We assume that the minimal encrypted dataset contributed by *s_i_* includes *ρ_i_* constraint factors, and all other parameters have the same meaning given above. Similarly, we can know the communication cost *E_Q_* for the query is as follows.

(13) $$\begin{array}{lll} E_{Q} & = & l_{id}+l_{t}+4\cdot l_{h}+n\cdot (l_{id}+l_{t})+l_{e}\cdot \sum_{i=1}^{n}\rho_{i}\\ & = & 4\cdot l_{h}+(n+1)\cdot (l_{id}+l_{t})+l_{e}\cdot \sum_{i=1}^{n}\rho_{i} \end{array}$$

In conclusion, we can finally obtain *E_total_*, which is the total communication cost for CSRQ, simply by adding *E_C_* to *E_Q_*. Then, we have

(14) $$\begin{array}{lll} E_{total} & = & E_{C}+E_{Q}\\ & = & \sum_{i=1}^{n}\left(l_{id}+l_{t}+(\delta_{i}-1)\cdot l_{h}+\delta_{i}\cdot l_{e}\right)\cdot L+4\cdot l_{h}+(n+1)\cdot (l_{id}+l_{t})+l_{e}\cdot \sum_{i=1}^{n}\rho_{i} \end{array}$$

In the next section, we will provide some further details about the performance analysis.

## 7. Experimental Results

For a further analysis of the performance of communication cost in CSRQ, we compare CSRQ with SafeQBloom, QuerySec, ESRQ and SecRQ by implementing these five schemes on a large real dataset from Intel Lab [26], and present the results of detailed performance evaluation obtained using MATLAB.

### 7.1. Contrastive Experimentfor Communication Cost

Since the sensor nodes in wireless sensor networks are mainly powered by battery, their energy is limited [27]. It is important to reduce the communication cost of sensor nodes in order to prolong the life of network. Thus, we analyze the communication costs of sensor nodes by comparing the performance of CSRQ with that of SafeQBloom, QuerySec, ESRQ and SecRQ on six aspects, including the network topology, the number of sensor nodes in a cell, the number of data items collected by a sensor node during a time slot, the length of an encoding data, an encrypted constraint factor and the number of constraint factors. We assume some default values of parameters, which are shown in Table 1 below.

**Figure 2:**
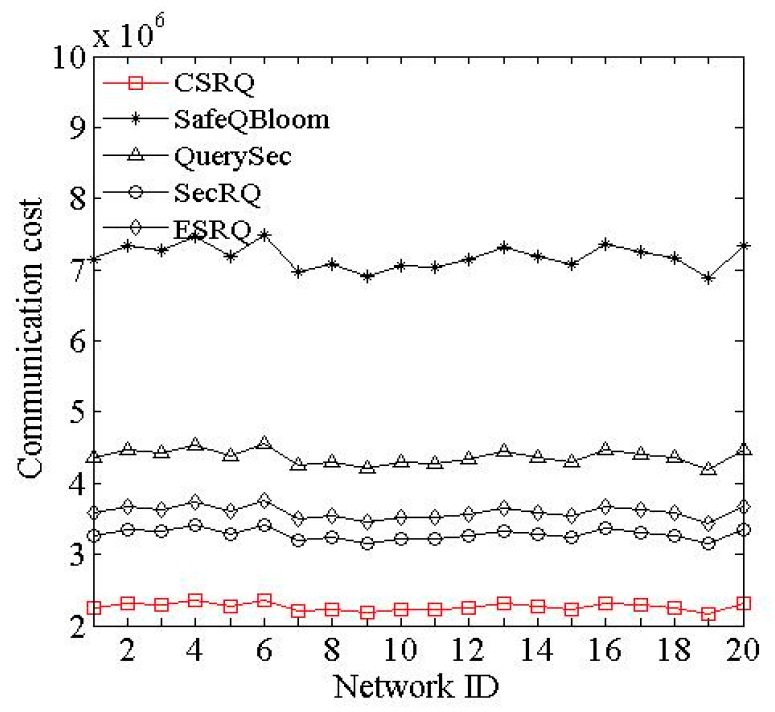
Impact of network ID on communication cost.

In our experiment, we assume that there are 20 groups of networks with various network topologies randomly distributed in the networks. The network ID of each group is unique. Then, the communication costs of query can be determined by computing the average costs of the 20 groups of networks. The details of experimental results and analysis are as follows:

(1) Impact of network ID. Figure 2 shows the communication cost of sensor nodes impacted by ID. Let other parameters be the default values. 

According to Figure 2, there is little change caused by different network topologies in these five mechanisms, and all of their communication costs of sensor nodes fluctuate within a small scope. The average communication cost for sensor nodes of SafeQBloom is relatively high, while the costs of CSRQ and SecRQ are lower. The communication cost of CSRQ is lowest, which is 68.5% lower than that of SafeQBloom and 9.4% lower than that of SecRQ. The reasons are as follows. In CSRQ, it contains *τ* −1 data items in each constraint factor except the first and last ones. Therefore, fewer messages will be submitted to *M* than those in SafeQBloom and SecRQ. What is more, during each time slot, the sensor nodes only need to send the minimal set of each factor’s upper boundary along with encrypted constraint chain to *M*, which can also reduce the communication cost of sensor node.

(2) Impact of *n* and *N*. We conducted experiment with different *n* and *N*, respectively, while other parameters are default values.

**Figure 3:**
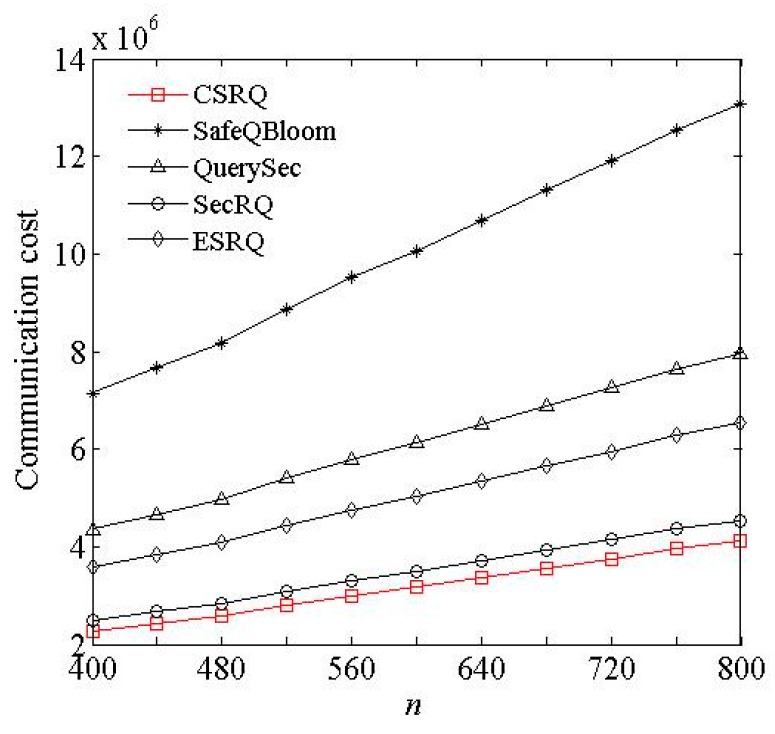
Impact of *n* on communication cost.

**Figure 4:**
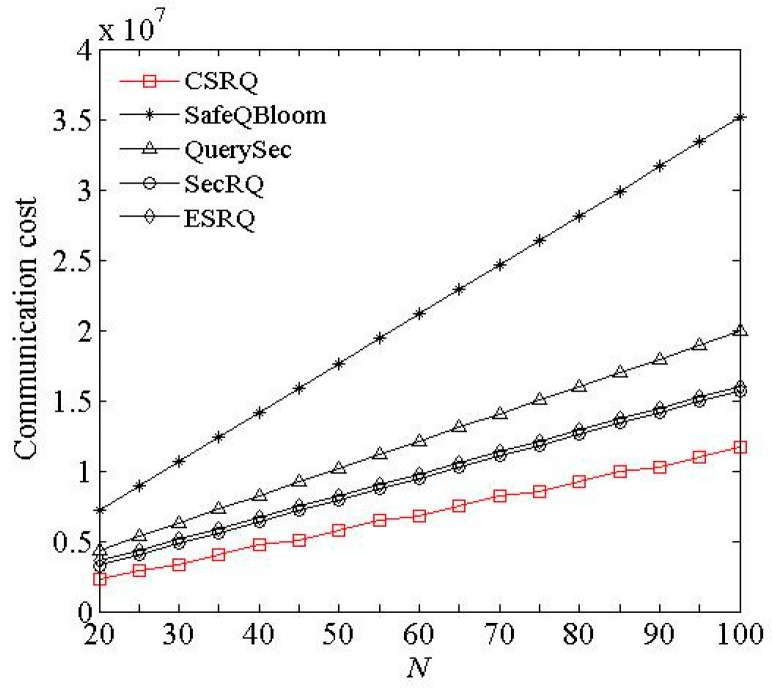
Impact of *N* on communication cost.

Figure 3 and Figure 4 show the communication cost of sensor nodes under the impact of *n* and *N*, respectively, where *n* is the number of sensor nodes in a cell, and *N* is the number of data items collected by a sensor node during a time slot. The communication cost of sensor nodes increases with *n* and *N* in these five schemes. In CSRQ, the 0-1 encoding scheme is used for comparison, which requires fewer messages to be transferred. It can significantly reduce the communication cost of sensor node. Thus, compared to other four schemes, CSRQ has the lowest communication cost of sensor nodes. In conclusion, the experimental data demonstrates that CSRQ can achieve security range query with lower communication cost than the existing security range query schemes.

**Figure 5:**
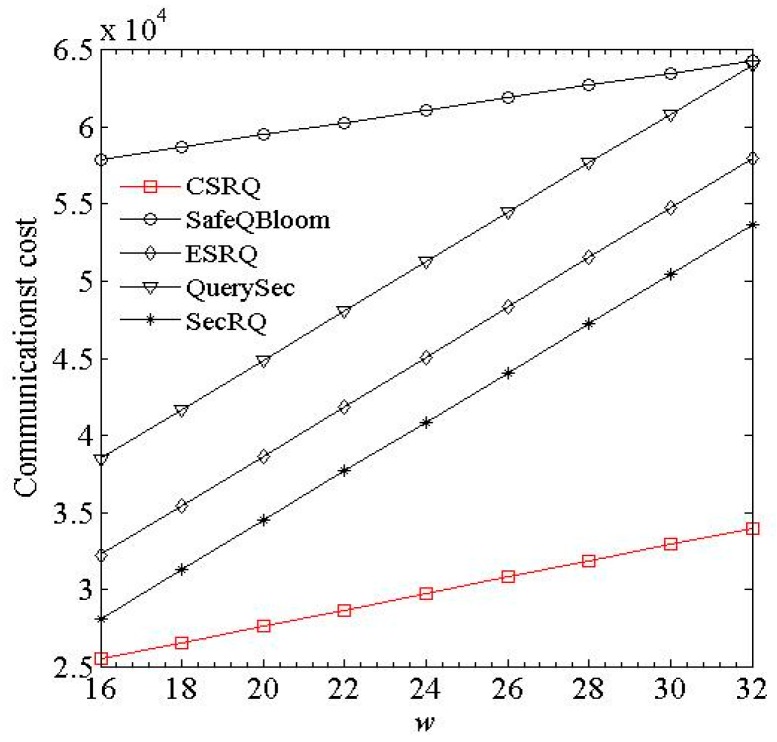
Impact of *w* on communication cost.

**Figure 6:**
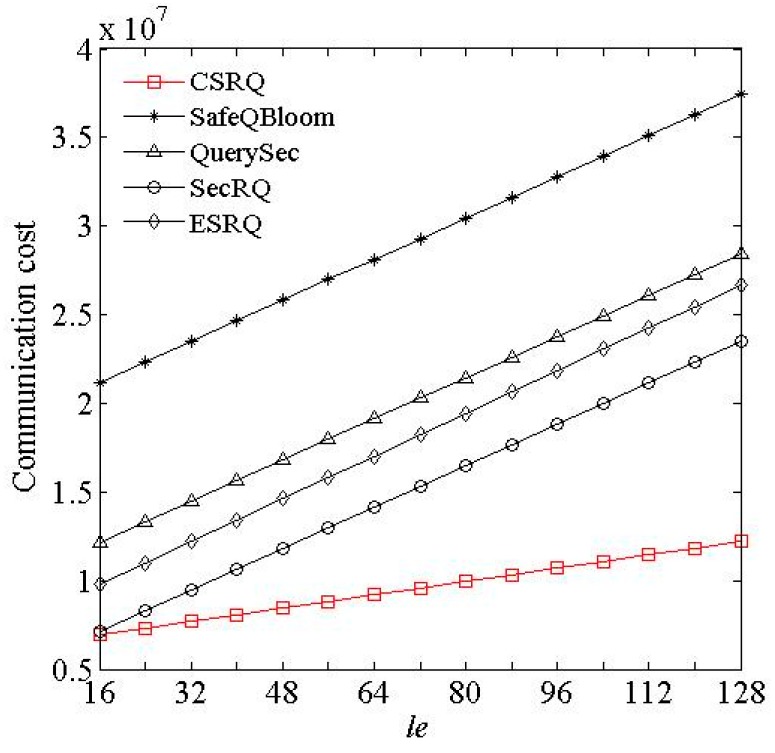
Impact of *l_e_* on communication cost.

(3) Impact of *w* and *l_e_*. As *w* and *l_e_* increase, the changes of communication costs of sensor nodes are shown in Figure 5 and Figure 6, where *w* denotes the bit length of data collected by sensor node, and *l_e_* denotes the average length of an encrypted constraint factor.

Figure 5 and Figure 6 show that in these five schemes, longer lengths of sensory data and encrypted constraint factor will both cause greater communication cost of sensor nodes. CSRQ has lower communication costs than the others. The reason is similar to Figure 2 and Figure 3.

**Figure 7:**
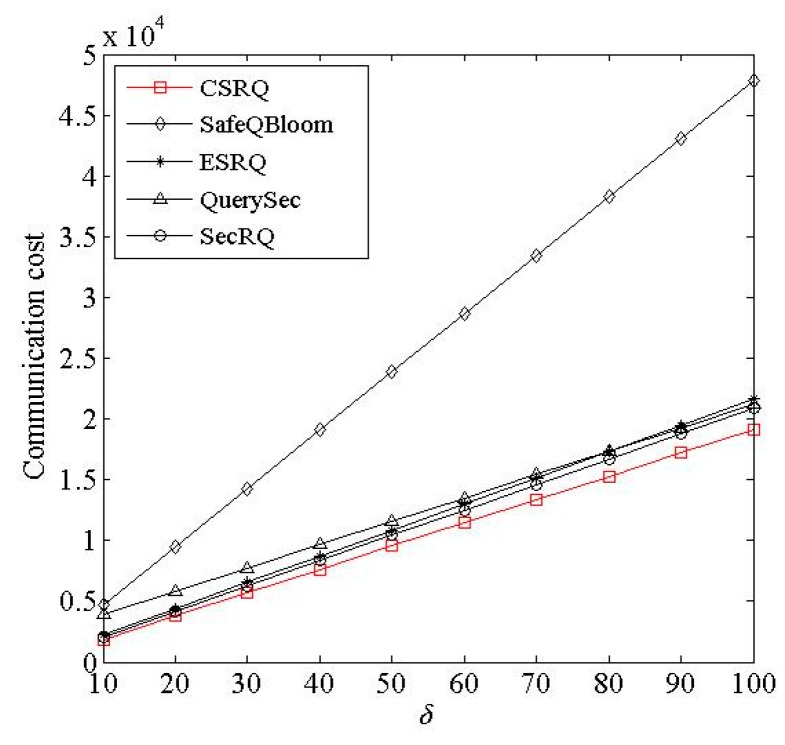
Impacted of *δ* on communication cost.

(4) Impact of *δ*. With ***δ*** changed, where ***δ*** denotes the number of encrypted constraint factor, the change of communication costs of sensor nodes is shown in Figure 7.

Figure 7 reveals that the sensor node’s communication costs in the five schemes increase with *δ*, and CSRQ has a lower communication cost than the others. The reason is as follow. The larger *δ* means that the sensor nodes submit more messages to *M*. In CSRQ, each sensor node only needs to submit *δ −* 1 minimal sets of boundary messages along with encrypted constraint chain to *M*. It means that fewer messages need to be transferred in CSRQ than in other schemes.

### 7.2. Contrastive Experiment for False Positive Rate

We define the false positive rate as the ratio of the number of unsatisfactory data items received by Sink to the number of data items satisfying query range. Therefore, the lower the false positive rate is, the higher the accuracy is.

**Figure 8:**
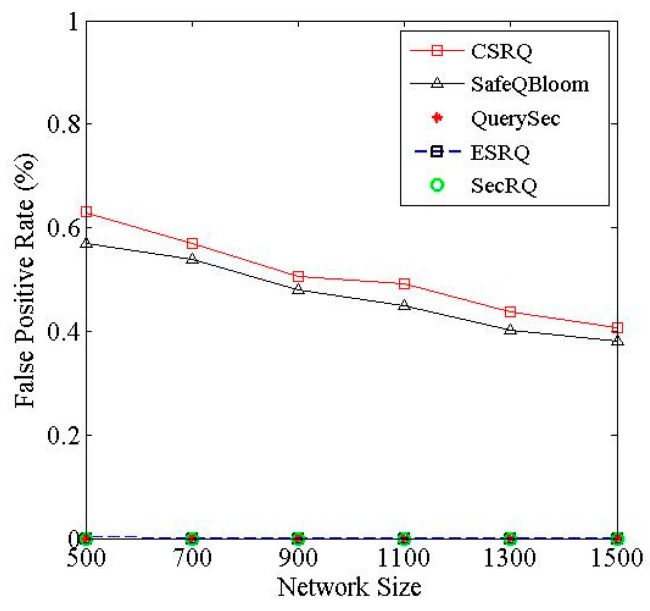
Impact of network size on false positive rate.

Figure 8 reveals the false positive rate impacted by the network size, where the network size refers to the data items collected by the sensor nodes and transmitted to *M*. We can see that QuerySec, ESRQ and SecRQ have no false positive, and the average false positive rates of SafeQBloom and CSRQ are 0.47% and 0.51%, respectively. In both CSRQ and SafeQBloom, the query results received by Sink may contain constraint factors in which only most parts of data items satisfy the query range. What is more, in CSRQ, it contains at most 2(*τ* − 1) unsatisfied data items in result received by Sink, and in general, *τ* > 2, which is a little more than that in SafeQBloom. Therefore, the false positive rate of former scheme is slightly higher than that of CSRQ. Furthermore, the false positive rate of CSRQ is very low, and it decreases with the increase of network size, and then gradually approaches 0. Thus, it has little effect on the performance of range queries.

The experiment results show that CSRQ provides a better performance in communication cost than current query protocols, such as SafeQBloom, QuerySec, ESRQ and SecRQ, in terms of efficiency.

## 8. Conclusions

In this paper, we propose CSRQ, a novel efficient protocol for processing range queries in two-tiered WSNs, which has great performance in privacy and integrity preservation. To preserve the privacy of data items in networks, we encrypt the data items collected by the sensor nodes through the encoding mechanisms. To preserve the integrity of query range and result, we present a novel encrypted constraint chain scheme to link data items collected by a sensor node to each other, which allows Sink to verify the integrity by checking the adjacent relations embedded in the encrypted constraint chains. The results of our experiment show that CSRQ has a better performance in terms of efficiency than current query protocols.

## Figures and Tables

**Table 1:** Experiment parameters.

Parameter	Value	Parameter	Value
The area covered networks/m^2^	80 × 80	Length of a time-slot/b	4 × 8
Radius of sensor node communication/m	10	Length of a sensor node ID/b	4 × 8
Number of collected data item in a time-slot (*N*)	20	Network IDs	20
Number of factor in a Constraint Chain (*τ*)	4	Length of a data (*w*)/b	16
Length of a constraint factor (*l_e_*)/b	128	Number of sensor nodes (*n*)	400
